# Case of Continued Priapism

**Published:** 1845-10

**Authors:** 


*Case of Continued Priapism.*—In the Western Journal of Medical and Physical Sciences a case was reported, about ten years since, similar to the following, from the London Lancet, except that the termination was in death.

R. W——, aged 26 years, a seaman, of stout make and florid complexion, stated that he arrived from Calcutta about the 12th of April, 1844, and had lodged with a female ever since. On Friday night, the 26th of the same month, he experienced an unusually fierce desire, with intense erection of the penis, which latter lasted throughout the night, with but little mitigation, being attended with pain of the left side of the organ, near the bulb, and an inclination of it towards the same side. These symptoms continuing, although congress was frequently resorted to, induced him to send for medical aid about 2 P.M. of the following day, when I found him in the condition above described.

On examination, the corpus spongiosum was found moderately turgid, especially the gland, which was situated near to the anterior superior spinous process of the ilium, and almost touching the abdominal parietes; the corpora cavernosa were fully distended and firm, scarcely yielding to pressure, and without any perceptible difference, either in color or firmness, at the part from which the inclination towards the left side commenced. When its restoration towards the median line was attempted, the pain was much increased. Beyond these he did not experience any uneasy sensation, nor was his health in any way deranged, the skin being rather moist, the tongue clean and moist, the pulse 75, full and soft, and the bowels open.

Let a cold lotion be applied, and let him take a quarter of a grain of tartar emetic, with one grain of powdered opium, every fourth hour; and three grains of calomel, with six of compound extract of colocynth, immediately.

April 28. Slightly improved, the pain and inclination towards the left side being lessened, and also the distention of the corpus spongiosum. Repeat the medicines. Nine P. M. The pain is slightly increased. Let six leeches be applied to the part.

29th. Decidedly better. The pain is much alleviated, and the corpora cavernosa rather flaccid. The organ has assumed the median line, and forms nearly a right angle with the abdomen. Repeat all the medicines.

30th. Much worse. The condition of the parts resembles that first described with the exception of the inclination to the left. On close questioning, he acknowledged having had frequent coitions since the 26th, and with the usual results, so that I advised his removal to the London Hospital. The report during the time he remained in the hospital was furnished by the senior dresser:—

R. W——, admitted for priapism, under the care of Mr. Luke, on the 30th of April, 1844. The corpora cavernosa are very much distended, but not the corpus spongiosum, or glans; the left crus of the penis is very firm.

Let him be bled to sixteen ounces, and twenty leeches be applied to the perineum. Apply a lotion of spirit in lime water, and give one scruple of calomel and rhubarb.

The pain was relieved by these means, but the tension of the parts was not at all diminished.

May 1st. About the same.

Let him have some house medicine, and saline antimonial mixture three times a day.

2d. Not at all relieved, the disease being as intense as ever.

Let him have two grains of calomel every third hour.

5th. The mercury has produced salivation, therefore, it is discontinued; the organ is not so tense; the angle which it it forms with the abdomen approaching nearer to a right angle.

10th. Left the hospital of his own accord; in fact much against the wishes of Mr. Luke. The penis is more flaccid, and forms a right, instead of an acute angle, with the pubis.

After leaving the hospital, he returned to his former abode, having free intercourse with the same female, until he left England for Sidney. During this time he had erections, and complete communication; the former proceeding to the usual extent, and afterwards gradually subsiding to the state in which the organ was previously to the venereal organism.

On leaving England (May 18th), his condition was as follows: Corpus spongiosum flaccid; corpora cavernosa moderately tense, and forming only an angle of about 45° with the pubis.

By letter, dated September, the condition last described remained without alteration for more than three months after he left England, but after that became rapidly palliated, and is now quite removed. On his return home he was perfectly cured, and without any ill results from his accident.

The remarks required by a case of this kind are but few. The lesion appears to have been caused by effusion of blood into the cells of the corpora cavernosa, which remained in a semi-fluid condition. The time (four months) during which it continued is very remarkable, whilst the perfect recovery of the patient eventually, seems to show that any instrumental mode of cure should not be attempted, unless undoubted signs of gangrene appear.

In a case treated by Mr. Calloway, and published in the *Medical Repository* for April, 1824, venesection, leeching, the warm bath, tobacco enemas, tartar emetic, and nitre, were exhibited and camphorated mercurial ointment was rubbed into the part, but without any benefit; wherefore, on the sixteenth day after the erection occurred, the left crus of the penis was punctured with a lancet, and a large quantity of dark grumous blood let out. By pressing the part, both corpora cavernosa were emptied through the aperture, which was followed for a few days by the escape both of pus and blood. The patient recovered, but never gained his power of erection, and therefore remained impotent for the remainder of his life, forming a marked contrast to the success of the foregoing case. In Mr. C.'s case, it occurred during, and was not diminished by repeated connexion.

